# Comparison of the Antibacterial Effect of Nano-calcium Hydroxide and Nano-chlorhexidine Against Enterococcus Faecalis: Short title: Antibacterial Effects of Nano-Calcium Hydroxide and Nano-CHX

**DOI:** 10.31661/gmj.v15i.4023

**Published:** 2025-06-17

**Authors:** Mamak Adel, Zohreh Asgari, Neda Hajihassani, Zohreh Bahrami, Mehdi Ranjbaran

**Affiliations:** ^1^ Department of Endodontics, Dental Cariers Prevention Research Center, Qazvin University of Medical Siences, Qazvin, Iran; ^2^ Department of Endodontics, School of Dentristry, Qavin University of Medical Sciences, Qazvin, Iran; ^3^ Department of Nanotechnology, Faculty of New Sciences and Technology, Semnan University, Semnan, Iran; ^4^ Non-communicanle Diseases Research Center, Research Institute for Prevention of Non-communicable Diseases, Qavin University of Medical Sciences, Qazvin, Iran

**Keywords:** Dentinal Tubule Disinfection, Enterococcus Faecalis, Intracanal Medicament, Nano-chlorhexidine Gel, Nano-calcium Hydroxide

## Abstract

**Background:**

Intracanal medicaments are used to eliminate residual microorganisms and provide a suitable environment for the healing of periapical tissues. The present study aimed to compare the antibacterial effect of nano-calcium hydroxide and nano-chlorhexidine as intracanal medicaments against Enterococcus faecalis.

**Materials and Methods:**

In this in vitro study, 170 single-rooted mandibular premolars were included. After crown removal, canal preparation was performed. Five teeth were randomly selected as the negative control group. Nano-intracanal medicaments were synthesized using the encapsulation method. Drug release tests were carried out, and particle sizes were determined using electron microscopy and dynamic light scattering analysis. Samples were inoculated with E. faecalis for 21 days. Subsequently, five teeth were randomly selected as the positive control group. The remaining samples were randomly divided into four groups of 40 teeth each (20 for day 3 and 20 for day 7). The intracanal medicaments used in each group were: calcium hydroxide, nano-Ca(OH)2-PLGA, 2% chlorhexidine gel, and 2% nano-chlorhexidine gel (MCM-41), respectively. After 3 and 7 days, microbial biofilm samples were collected, and bacterial colony-forming units (CFU) were counted. Two-way ANOVA was used to compare the mean CFU counts among the groups at different time intervals. The significance level was set at 0.05.

**Results:**

Based on statistical analysis, on day 3, the 2% nano-chlorhexidine gel (MCM-41) and nano-Ca(OH)2-PLGA groups showed a significantly greater reduction in CFU/ml (P0.05) compared to the other groups. On day 7, the 2% nano-chlorhexidine gel (MCM-41) and 2% chlorhexidine gel groups demonstrated significantly greater reductions in CFU/ml (P0.05) than the remaining groups. Over time, the antibacterial efficacy of the nano-formulations declined, whereas the effectiveness of the 2% chlorhexidine gel and calcium hydroxide increased.

**Conclusion:**

According to the findings of the present study, nano-formulated medicaments were more effective in eliminating E. faecalis biofilm from the root canal only in short-term.

## Introduction

Studies have demonstrated that the outcome of root canal treatment is mainly dependent on the quality of chemomechanical canal preparation and the extent to which bacteria, their byproducts, and necrotic tissue are eliminated during this process. Of the various microorganisms implicated in treatment failure, one species is of particular concern due to its resilience. Enterococcus faecalis is a facultative anaerobic coccus capable of surviving under harsh conditions due to its unique ability to invade dentinal tubules, form biofilms, and endure nutrient deprivation [1]. It has been identified as a dominant microorganism in 22% to 77% of endodontic treatment failures, often as the sole or principal component of the microbial flora. Consequently, E. faecalis plays a key role in the etiology of periapical lesions and persistent infections following root canal therapy [2].

Although chemomechanical preparation of the root canal significantly reduces the microbial load, it does not eliminate all microorganisms [3]. This limitation is exacerbated by anatomical complexities. The complex morphology of the root canal system, including numerous ramifications and anatomical irregularities, hinders thorough cleaning and chemomechanical preparation, which may result in treatment failure or persistent endodontic infection [4]. Therefore, to maximize disinfection of the infected root canal system, particularly in necrotic teeth and retreatment cases, the use of intracanal medicaments is essential to effectively eradicate residual microorganisms inaccessible to endodontic instruments [5].

Calcium hydroxide [Ca(OH)₂] is commonly used as an intracanal medicament [6]. Its antimicrobial activity is attributed to its high pH (approximately 12), which leads to bacterial cell wall disruption through the release of hydroxyl ions [7]. For optimal effectiveness, calcium hydroxide must come into direct contact with microorganisms. However, due to its relatively large particle size, it cannot penetrate deeply into dentinal tubules, resulting in limited efficacy against certain microorganisms, such as E. faecalis [8]. Additionally, the buffering capacity of the dentinal matrix diminishes its disinfecting potential [9]. Consequently, despite its broad-spectrum antibacterial activity as an intracanal medicament, some studies have reported a reduced effectiveness of calcium hydroxide against specific microorganisms, such as E. faecalis, Streptococcus sanguis, and Candida albicans, which may remain viable within dentinal tubules [10]. The buffering action of dentin prevents the medicament from maintaining the high pH levels necessary for its bactericidal effect, especially in the deeper layers of dentin where E. faecalis often resides [11]. It is hypothesized that modifying the physical form of this medicament could overcome these barriers. Theoretically, calcium hydroxide nanoparticles (NCH) have a greater potential to penetrate deeper dentinal layers due to their smaller size and higher surface area, and may therefore exert a more effective antibacterial action against E. faecalis.

Another widely used antibacterial agent in endodontics is chlorhexidine. Chlorhexidine (CHX) is a cationic bis-biguanide compound with broad-spectrum antibacterial activity against both Gram-positive and Gram-negative bacteria [12]. Its positively charged molecules interact with the negatively charged phosphate groups on microbial cell walls, leading to membrane disruption, leakage of cytoplasmic contents, and eventual cell death [13]. A 2% concentration of chlorhexidine is commonly used as an intracanal medicament in endodontics [12]. It has shown efficacy in eliminating microorganisms such as E. faecalis, which are resistant to calcium hydroxide. Due to the possibility of chlorhexidine extruding beyond the apical foramen, its application in a viscous gel form is considered more suitable for intracanal use [13]. The advancement of nanotechnology offers promising avenues to enhance the properties of such medicaments. Studies have shown that nanoparticles, due to their higher surface area-to-volume ratio, increased surface charge density, controlled release profiles, and unique biological properties, can enhance the therapeutic effects of drugs. They offer superior antibacterial performance through improved penetration and accessibility, while also reducing cytotoxicity, drug dosage, and overall treatment cost [14].

Recent research supports the potential of nanomaterial-based intracanal medicaments. Parolia et al. [15] reported that chitosan-propolis nanoparticles (CPNs), when used as intracanal medicaments, may be effective in reducing E. faecalis in failed root canal treatments. Similarly, Balto et al. [16] demonstrated that the application of nanoparticles as intracanal medicaments can be practical against E. faecalis. The findings of Afkhami et al. [17] indicated favorable antibacterial effects of DAP and TAP formulations, as well as a combination of calcium hydroxide with silver nanoparticles, after one week of application. Building upon this foundation, given the critical importance of maximizing biofilm elimination from the root canal system and the clinical relevance of E. faecalis as a resistant pathogen in endodontic infections, along with the need for new intracanal medicaments capable of disrupting biofilm structures, the present study was designed to compare the antibacterial effects of nano-calcium hydroxide and 2% nano-chlorhexidine against biofilms formed by E. faecalis, in comparison with conventional intracanal medicaments. To this end, in this study, nano-calcium hydroxide was prepared using poly(lactic-co-glycolic acid) (PLGA) nanoparticles and used as an intracanal medicament. In addition, mesoporous silica nanoparticles (MSNs) were synthesized using MCM-41 as a carrier, and 2% chlorhexidine was subsequently loaded into these nanostructures.

## Materials and Methods

This study was an in vitro experiment, registered under the ethical approval code IR.QUMS.REC.1403.024 on 11/02/1403 (Persian calendar) at Qazvin University of Medical Sciences. A total of 170 extracted, single-rooted, single-canal mandibular premolars from adult patients were selected. The included teeth had round root canal cross-sections and were free from resorption, calcification, and severe curvature. Exclusion criteria included the presence of dental cracks, internal or external resorption, calcifications within the canal or pulp chamber, severe root or canal curvature, and open apices. Based on previous studies and using G*Power software , with an alpha level of 0.05, a study power of 0.80, four experimental groups at two different time points, and an effect size of 0.38, the sample size was calculated. Using a consecutive purposive sampling method, 20 mature single-canal premolars were required per group at each time point, along with five teeth each for the positive and negative control groups.

### Sample Preparation

A total of 170 single-rooted, mature mandibular premolars without resorption, calcification, or severe canal curvature were collected (radiographs were taken to assess canal conditions). Teeth were examined under a stereomicroscope to ensure the absence of cracks. The collected teeth were initially stored in 0.5% chloramine-T solution for one week and then transferred to sterile saline at room temperature. Crowns were sectioned using a low-speed handpiece and diamond disc (Dentsply Maillefer, Ballaigues, Switzerland) to standardize root lengths to 12 mm. Working length was established by inserting a size #15 K-file (Mani, Tochigi, Japan) into the canal until it was visible at the apical foramen, then subtracting 0.5 mm. Root canals were instrumented to the working length using the single-length technique with Protaper files (S1 to F3; Dentsply Maillefer, Ballaigues, Switzerland) under irrigation with 2 mL of 5.25% sodium hypochlorite (NaOCl) (Hyponic, NikDarman Co., Tehran, Iran). To remove the smear layer, the canals were sequentially irrigated with 2 mL of 17% EDTA (Morvabon, Iran) and 2 mL of 5.25% NaOCl, each for 2 minutes. Intermediate and final rinses were performed with 5 mL of normal saline (SAMEN Co., Iran) (15). Apical foramina were sealed with self-cure glass ionomer (GC, Tokyo, Japan), and root surfaces were coated with two layers of nail varnish. The teeth were then sterilized in an autoclave (LTE, Oldham, Lancashire, UK) at 121°C for 20 minutes. Each tooth was transferred into a sterile Eppendorf tube containing brain-heart infusion (BHI) broth (Merck, Darmstadt, Germany). The tubes were then sealed and incubated at 37°C for 48 hours (Memmert UF 55).

Five teeth were randomly selected as the negative control group to verify the accuracy of the sterilization process. These samples were incubated in BHI broth for 24 hours, and the absence of bacterial growth confirmed the lack of microbial contamination.

### Preparation of Nano-formulated Calcium Hydroxide

To prepare the nano-formulated calcium hydroxide [Ca(OH)2], the solvent displacement method was employed [18]. The encapsulation efficiency (EE) of calcium hydroxide in PLGA nanoparticles was determined indirectly by measuring the concentration of the free drug in the dispersion medium [19].

### Drug Release Assessment

Drug release profiles from Ca(OH)2-PLGA nanoparticles and conventional Ca(OH)2 in phosphate-buffered saline (PBS) were assessed using the direct dialysis bag technique over a period of one week [20].

### Synthesis and Drug Loading for MCM-41 Nanoparticles

MCM-41 nanoparticles were synthesized according to established protocols [20]. For loading chlorhexidine into the nanogel matrix, an encapsulation technique was utilized. Specifically, a 2% chlorhexidine solution was added to the MCM-41 nanoparticles to produce a 2% nano-chlorhexidine gel. To determine drug loading efficiency, the resulting mixture was stirred at room temperature for 24 hours. Following this incubation period, the nanogel was separated via centrifugation (Farzaneh Arman - HS 18500 R) at 10,000 rpm for 10 minutes at 4°C. The drug loading percentage was calculated using subsequently detailed equations.

Drug loading (%) ="Amount of chlorhexidine loaded in nanoparticles" / "Total weight of nanoparticles" ×100

Encapsulation efficiency (%) =

("Total amount of drug added" - "Amount of drug in the solution phase" )/"Total amount of drug added" ×100

### Calibration Curve Construction

**Figure-1:**
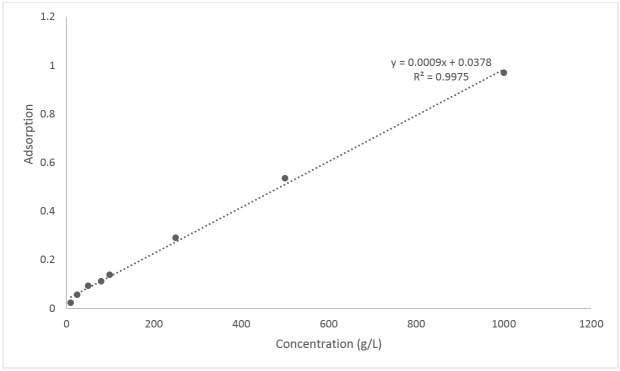
Figure 1. Calibration curve and linear regression equation obtained for standard chlorhexidine solutions

To determine the amount of chlorhexidine loaded and subsequently released at each time interval, a calibration curve was first constructed. To prepare the stock solution, a proportional dilution was performed: 5 µL of the primary solution [20% chlorhexidine (20 g in 100 mL)] was transferred using a micropipette and diluted to a final volume of 99.995 mL. For calibration, standard solutions of chlorhexidine were prepared at concentrations of 10, 25, 50, 80, 100, 250, 500, and 1000 mg/L. Using the solution with the lowest concentration, the maximum absorbance wavelength was determined via a UV-Visible spectrophotometer and was found to be 252 nm. The absorbance of all standard solutions was then measured at this wavelength. A calibration curve of absorbance versus concentration was plotted for these standards. Based on the Beer-Lambert law, which states that absorbance is directly proportional to concentration, a linear graph with a correlation coefficient (R²) of 0.9975 was obtained, indicating a strong linear fit. The resulting linear equation was used to calculate the chlorhexidine loading and release values. Figure-1 presents the calibration curve and the corresponding linear equation. The percentage and efficiency of chlorhexidine loading were calculated according to standard equations.

### Drug Release Assessment Protocol

To evaluate drug release from the delivery system, an acetate buffer with a pH of 5 was prepared. For activation, dialysis bags were first immersed in a boiling 5% (w/w) sodium bicarbonate solution for 15 minutes. They were then removed and rinsed with distilled water, repeating this rinse three times. Subsequently, the dialysis bags were placed in a boiling 5% (w/w) EDTA solution for 15 minutes. After the designated time, the bags were removed and again washed with distilled water, with the wash cycle repeated three times. Finally, the prepared dialysis bags were stored in 20% ethanol at 4°C until use.

Based on the drug’s saturation solubility and kinetic requirements, a final volume of 50 mL was selected. Accordingly, 50 mL of acetate buffer was poured into each test container. Three dialysis bags, each measuring 10 cm in length, were prepared and filled with 5 mg (equivalent to 500 µL) of MCM-41 nano-chlorhexidine gel. At predetermined time intervals, 1 mL aliquots were withdrawn for UV-Visible spectrophotometric analysis at a wavelength of 260 nm. An equal volume of fresh acetate buffer was then added to maintain a constant total volume. Drug release was monitored at 37°C and pH 5. Sampling was continued until absorbance values reached a plateau. To quantify drug release over time, the percentage release was plotted against time. The percentage of drug released at each time point was calculated using the following formula:

Percentage Release (%) = (Drug concentration at each time point / Total drug concentration) × 100

### Nanoparticle Characterization

The morphology of the synthesized drug-loaded nanoparticles was examined using scanning electron microscopy (SEM) (ZEISS Sigma 300). Energy-dispersive X-ray spectroscopy (EDS), an integrated module within the SEM system, was employed to determine the elemental composition of the solid samples. To assess the surface charge of the nanoparticles, expressed as zeta potential, laser Doppler electrophoresis was performed using the M3-PALS system integrated into the Zetasizer Nano ZS.

X-ray diffraction (XRD) analysis was conducted using a PHILIPS PW1800 (Germany) instrument operated at 40 kV and 30 mA, with Cu Kα radiation. Diffraction patterns were recorded across a 2θ range of 10° to 90° to identify crystal phases and structural features.

Fourier-transform infrared (FTIR) spectroscopy was used to identify functional groups. Spectral data were collected in the range of 400-4000 cm-¹ for sample characterization.

### Antimicrobial Activity Assay

The minimum inhibitory concentration (MIC) and minimum bactericidal concentration (MBC) of all four formulations against Enterococcus faecalis (ATCC 29212) were independently determined using the microbroth dilution method (microtiter assay in a 96-well plate) [21].

All microbiological procedures were carried out under a Class II laminar flow biological safety hood. Enterococcus faecalis (ATCC 29212) was cultured on Todd Hewitt agar plates and incubated at 37°C for 24 hours to obtain isolated colonies. Single colonies were then inoculated into 10 mL of BHI broth and incubated at 37°C for 24 hours. The bacterial suspension was subsequently standardized to 1.5 × 108 CFU/mL (equivalent to a 0.5 McFarland standard). A volume of 0.01 mL of the prepared suspension was injected into the root canals using an insulin syringe. The samples were incubated under anaerobic conditions at 37°C for three weeks, with the culture medium being replaced daily. Following the incubation period, five teeth were randomly selected as the positive control group, and microbial cultures were performed. The remaining teeth were randomly divided into four experimental groups, each consisting of 40 specimens (20 specimens for day 3 and 20 for day 7), for intracanal drug application.

The first group received calcium hydroxide paste (Nik Darman, Iran); the second group was treated with nano-Ca(OH)2-PLGA; the third group received 2% chlorhexidine gel (Clorex Gel 2%, Nik Darman, Iran); and the fourth group was treated with 2% nano-chlorhexidine gel (MCM-41-based). To prepare the calcium hydroxide and nano-calcium hydroxide pastes, 1 mg of powder was mixed with 1 mL of normal saline to obtain a paste with a final concentration of 1 mg/mL. The prepared pastes were delivered into the root canals using a size #30 Lentulo spiral (Mani, Tochigi, Japan) [22]. The 2% chlorhexidine gel and 2% nano-chlorhexidine gel were injected into the canals using a sterile 27-gauge syringe (AVA, Semnan, Iran) until complete canal filling was achieved. The coronal orifices were then sealed with paraffin wax. All specimens were incubated at 37°C for experimental periods of 3 and 7 days. At the end of days 3 and 7, canals were dried with sterile paper points, and dentin sampling was performed using a sterile #5 Gates Glidden drill (Mani, Tochigi, Japan; 1.3 mm diameter) mounted on a low-speed handpiece [23]. To standardize the volume of dentin debris collected, only a single stroke was applied per sample. The dentin shavings were transferred into microtubes containing 1 mL of sterile Tryptic Soy (TS) broth and incubated under anaerobic conditions at 37°C for 24 hours. After incubation, the contents of each tube were serially diluted five times (100 µL into 100 µL sterile saline per dilution). Then, 50 µL of each diluted sample was transferred to TS agar plates using a micropipette and incubated at 37°C for 24 hours. Finally, bacterial colonies were counted under a biological safety hood using magnification, applying the appropriate dilution factor to ensure accuracy. The counting process was performed in a blinded manner to ensure objectivity and impartiality.

### Data Analysis

Data collection and Statistical analysis was performed using SPSS version 25 (IBM Corp., Armonk, NY, USA). Descriptive statistics were expressed as mean and standard deviation. Two-way ANOVA was applied to compare the mean colony-forming units (CFU) among the groups across different time intervals. The normality of data distribution was assessed using histogram plots and the Kolmogorov-Smirnov test. A significance level of 0.05 was considered for all statistical tests.

## Results

**Figure-2:**
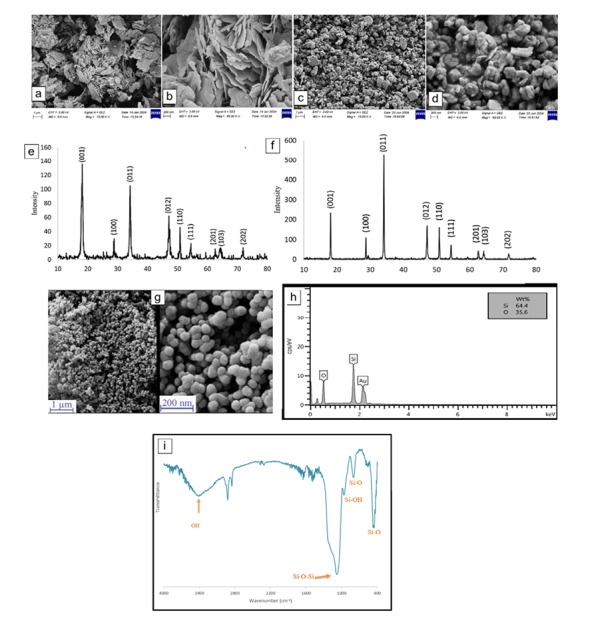
Figure 2. Physicochemical characterization of the formulations:
(a) SEM image of Ca(OH)2–PLGA nanoparticles (1 μm), (b) SEM image of Ca(OH)2–PLGA nanoparticles (200 nm), (c) SEM image of Ca(OH)2 particles (1 μm), (d) SEM image of Ca(OH)2 particles (200 nm), (e) XRD patterns of Ca(OH)2–PLGA (f) XRD patterns of Ca(OH)2, (g) SEM image of MCM-41–based chlorhexidine nanogel (1 μm and 200 nm), (h) EDS spectrum of MCM-41 nanoparticles, (i) FTIR spectrum of MCM-41–based chlorhexidine nanogel.

**Figure-3:**
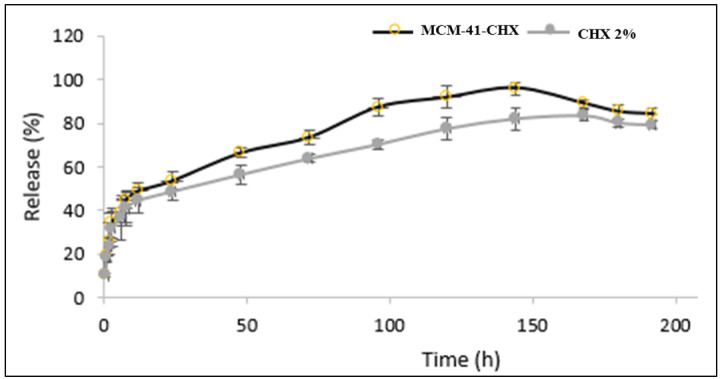
Figure 3. Drug release profiles

**Table:** Table1. Comparative Physicochemical Properties of Nanoformulations

**Formulation**	**EE (%)**	**DL (%)**	**Particle morphology**	**Particle size (nm)**	**Zeta potential (mV)**	**Crystallite size (nm)**
Ca(OH) _2_	—	—	Irregular	200-600	—	~45
Ca(OH)_2_-PLGA	70 ± 0.04	63 ± 0.05	Plate-like	100-300	−13	~20
2% Chlorhexidine gel	—	—	Amorphous	—	—	—
MCM-41-CHX nanogel	89 ± 0.05	75 ± 0.06	Spherical	70-80	+27	—

**Table:** Table2. Mean CFU/ml of Experimental Groups

**Experimental Groups **	**Day 3 - Mean CFU/ml ± SD **	**Day 7 - Mean CFU/ml ± SD **
Ca(OH)_2_-PLGA	79.65 ± 12.991	100.65 ± 14.460
Calcium Hydroxide	165.95 ± 25.120	151.40 ± 28.799
2% Chlorhexidine Nano-Gel (MCM-41)	44.25 ± 8.759	61.30 ± 10.428
2% Chlorhexidine Gel	121.10 ± 16.905	92.60 ± 18.483

### Physicochemical Characterization of the Formulations

The physicochemical characteristics of the nanoformulated systems were evaluated and compared with their corresponding conventional formulations. Encapsulation efficiency and drug loading capacity demonstrated effective incorporation of active agents within the nanocarriers. The Ca(OH)2-PLGA nanoparticles exhibited an encapsulation efficiency of approximately 70% and a drug loading of 63%, whereas the MCM-41-based chlorhexidine nanogel showed higher values, reaching approximately 89% and 75%, respectively. These quantitative parameters, together with particle size, morphology, surface charge, and structural characteristics, are summarized in Table-1.

Zeta potential measurements indicated distinct surface charge characteristics among the formulations (Table-1). Ca(OH)2-PLGA nanoparticles showed a negative zeta potential of approximately −13 mV, suggesting moderate colloidal stability. In contrast, the MCM-41-based chlorhexidine nanogel exhibited a positive surface charge of approximately +27 mV, indicative of improved electrostatic stabilization and reduced aggregation tendency. Morphological evaluation by scanning electron microscopy revealed pronounced differences between nanoformulated and non-nano materials (Figure 2a-d). Conventional Ca(OH)2 particles showed irregular morphology with a broad and heterogeneous size distribution in the range of approximately 200-600 nm. In contrast, Ca(OH)2-PLGA nanoparticles exhibited a well-defined plate-like morphology with lateral dimensions of approximately 100-300 nm and thicknesses of about 20-30 nm (Figure-2a,b), indicating successful morphological modification through polymeric encapsulation.

Structural analysis by X-ray diffraction confirmed that both Ca(OH)2-PLGA nanoparticles and conventional Ca(OH)2 retained a crystalline hexagonal structure consistent with the standard reference pattern (JCPDS No. 01-072-0156), demonstrating that the encapsulation process did not alter the intrinsic crystal phase (Figure-2e,f). A notable increase in the intensity of the (001) diffraction peak was observed for Ca(OH)2-PLGA nanoparticles compared with Ca(OH)2, suggesting preferential crystal growth along this plane. Crystallite size calculations revealed smaller crystallite dimensions for Ca(OH)2-PLGA nanoparticles (~20 nm) relative to Ca(OH)2(~45 nm), which is consistent with the reduced particle dimensions observed in SEM images.

The MCM-41-based chlorhexidine nanogel displayed uniform spherical nanoparticles with relatively narrow size distribution and average diameters of approximately 70-80 nm, arranged in clustered assemblies (Figure-2g).

Compositional and chemical structure analyses further supported successful nanoformulation. The EDS spectrum of the MCM-41 nanoparticles confirmed the presence of only silicon and oxygen, indicating high material purity (Figure-2h). FTIR spectroscopy of the MCM-41-based chlorhexidine nanogel revealed characteristic absorption bands corresponding to Si-O-Si, Si-O, and silanol (Si-OH) groups, along with C-H stretching vibrations associated with the incorporated organic component, confirming successful integration within the mesoporous silica framework without structural degradation (Figure-2i).

### In Vitro Drug Release Profiles

The cumulative drug release profiles of Ca(OH)2-PLGA nanoparticles versus free Ca(OH)2 and 2% chlorhexidine MCM-41 nanogel versus conventional 2% chlorhexidine gel are shown in Figure-3. Ca(OH)2-PLGA nanoparticles exhibited a pronounced initial burst release, reaching approximately 95% within the first 12 hours, followed by a gradual decrease in release rate until day 3 and a relatively stable sustained release thereafter. This initial burst can be attributed to drug adsorbed on or near the nanoparticle surface. In contrast, free Ca(OH)2 showed a lower initial release (28% at 12 hours) and consistently lower cumulative release throughout the study period. Both chlorhexidine formulations demonstrated an initial burst release within the first 12 hours (44% for conventional gel and 48% for nanogel). Subsequently, the nanogel exhibited a more sustained and higher cumulative release, reaching approximately 96% by day 6, whereas the conventional gel reached approximately 83% by day 7.

### Antibacterial Activity

The antibacterial activity against Enterococcus faecalis (ATCC 29212) demonstrated superior efficacy of the nano-formulations. The 2% chlorhexidine MCM-41 nanogel showed the lowest MIC (6.25 μg/mL) and MBC (12.5 μg/mL) values compared with the conventional chlorhexidine gel (MIC: 25 μg/mL, MBC: 55.5 μg/mL). Similarly, Ca(OH)2-PLGA nanoparticles exhibited lower MIC (15.6 μg/mL) and MBC (31.2 μg/mL) values than free Ca(OH)2 (MIC: 62.5 μg/mL, MBC: 125 μg/mL).

The mean CFU count of the positive control group was 182 CFU/mL, whereas the negative control group showed near-zero CFU values. Two-way ANOVA revealed a significant difference in CFU counts among treatment groups (P<0.001), while the effect of time was not statistically significant (P=0.665). A significant interaction between treatment group and time was observed (P<0.001).

The mean CFU values for each experimental group at days 3 and 7 are summarized in Table-2. On day 3, the 2% chlorhexidine MCM-41 nanogel and Ca(OH)2-PLGA groups demonstrated the greatest reduction in CFU counts compared with other groups (P<0.05). On day 7, the lowest CFU values were observed in the 2% chlorhexidine MCM-41 nanogel and conventional chlorhexidine gel groups. No statistically significant difference was observed between Ca(OH)₂-PLGA and conventional chlorhexidine gel on day 7 (P=0.554), whereas all other comparisons were significant (P<0.001).

## Discussion

Although a polymicrobial biofilm would be more appropriate for evaluating the antibacterial efficacy of intracanal medicaments, similar to previous studies, a monospecies biofilm of E. faecalis (ATCC 29212) was used in the present investigation to enable more reliable and valid comparisons with other established studies [24]. A bacterial suspension equivalent to the 0.5 McFarland standard was employed, consistent with the methodology reported by Asnaashari and Batinic [25]. Antibacterial activity was assessed using the colony-forming unit (CFU) count method, as it is the most commonly utilized technique in similar research [7]. In previous studies, calcium hydroxide has been shown to eliminate E. faecalis at a depth of 100 µm. However, its antibacterial efficacy markedly declines at a depth of 200 µm [26]. The limited solubility of calcium hydroxide in vitro, its restricted diffusion capacity within dentinal tubules, and the inherent buffering effect of dentin are considered major obstacles to achieving the alkaline pH levels necessary to eliminate bacteria at greater dentinal depths [27].

The findings of studies by Gomes et al. [12] and Krithikadatta et al. [28] demonstrated that 2% chlorhexidine gel exhibited superior antibacterial activity compared to 0.2% chlorhexidine gel or calcium hydroxide combined with 0.2% chlorhexidine gel. Given its potent antibacterial effects against E. faecalis, 2% chlorhexidine gel was selected for use in the present study. The encapsulation process results in particles smaller than the diameter of dentinal tubules, thereby enhancing the penetration of the antibacterial agent into these tubules. Similarly, the study by Seneviratne et al. showed that nanoparticle-encapsulated chlorhexidine, under in vitro conditions, could significantly reduce the activity of planktonic bacteria and monospecies biofilms such as Streptococcus mutans, Streptococcus sobrinus, Fusobacterium nucleatum, and Enterococcus faecalis [29]. Furthermore, numerous studies have reported that nanogels possess advantageous properties, such as high loading capacity for hydrophilic drugs. Their network structure protects the encapsulated drug molecules from degradation, as enzymes are unable to penetrate the particles [30].

In the present study, the mean particle sizes of the 2% chlorhexidine nanogel (MCM-41) and nano-Ca(OH)2-PLGA were 75 nm and 182 nm, respectively. These findings are consistent with previous research aimed at leveraging nanotechnology to develop improved antibacterial intracanal medicaments [31]. Factors such as zeta potential and polydispersity index may also influence the antibacterial efficacy of a compound. Zeta potential refers to the electrostatic potential at the particle surface, which strongly influences nanoparticle stability. Typically, stabilized nanoparticles are expected to exhibit a zeta potential of approximately ±30 millivolts to maintain a well-dispersed and stable suspension, thereby preventing rapid particle aggregation [32]. In the current study, the zeta potentials of the 2% chlorhexidine nanogel (MCM-41) and nano-Ca(OH)2-PLGA were measured at +27 mV and −13 mV, respectively. These values fall within the range reported in previous studies [33]. Our results indicate that the 2% chlorhexidine nanogel (MCM-41) and nano-Ca(OH)2-PLGA showed the most significant reduction in bacterial colony-forming units (CFUs) compared to conventional 2% chlorhexidine gel and calcium hydroxide. This outcome supports the notion that smaller particles, due to their larger surface-area-to-volume ratio, exhibit higher reactivity [34].

The release profiles of all four medications demonstrated sustained drug release from day one to day seven. The release rate of the nanoformulations was higher compared to that of their conventional counterparts. This enhanced release from nano-drugs can be attributed to their smaller particle size and greater solubility. In contrast, the higher molecular weight and degree of cross-linking in conventional drug forms increase viscosity and reduce the rate of drug release. Specifically, as the polymer content increases, the resulting gel becomes more solidified, hindering water penetration and thereby decreasing drug diffusion [35].

In the present study, the synthesized 2% chlorhexidine nanogel (MCM-41) and nano-Ca(OH)2-PLGA exhibited encapsulation efficiencies of 89% and 70%, respectively, and drug loading capacities of 75% and 63%. The minimum inhibitory concentration (MIC) and minimum bactericidal concentration (MBC) for the 2% chlorhexidine nanogel (MCM-41) were determined to be 6.25 µg/mL and 12.5 µg/mL, respectively. For conventional 2% chlorhexidine gel, MIC and MBC values were 25 µg/mL and 55.5 µg/mL, respectively. The MIC and MBC of nano-Ca(OH)2-PLGA were found to be 15.6 µg/mL and 31.2 µg/mL, respectively, whereas calcium hydroxide showed MIC and MBC values of 62.5 µg/mL and 125 µg/mL, respectively. Since MIC and MBC values are determined under in vitro conditions, where bacteria exist in planktonic form—unlike the clinical setting, where they predominantly form biofilms and encounter interfering factors such as dentinal buffering—higher concentrations, as commonly used in clinical applications, were employed. The MIC and MBC results obtained in this study align with previous findings, indicating that intracanal nanoformulations are effective at significantly lower concentrations compared to their conventional counterparts.

Based on the findings of the present study, the superiority of nano-formulated medicaments over conventional forms in eliminating E. faecalis from root canals is evident. Numerous studies have reported similar results [36]. Furthermore, a three-day intracanal medication regimen using the nanoformulations investigated in this study may yield favorable outcomes. Nevertheless, further research involving varied treatment durations is necessary to establish definitive conclusions. In line with the current results, a study by Tulu et al. [37] also demonstrated that the combination of silver nanoparticles with chlorhexidine significantly reduced bacterial counts compared to other medicaments at both 1-day and 7-day application intervals. Additionally, the combination of silver nanoparticles with calcium hydroxide outperformed calcium hydroxide alone at both time points, further supporting the enhanced efficacy of nanoparticle-based therapies.

In a study conducted by Sireesha et al. [38], the depth of penetration and antibacterial properties of calcium hydroxide, nano-calcium hydroxide, chitosan, and nano-chitosan were compared as intracanal medicaments. The results indicated that nano-formulated intracanal drugs not only exhibited greater dentinal tubule penetration but also demonstrated more potent antibacterial activity. More recently, Firas et al. [39] investigated the antibacterial efficacy of nano-calcium hydroxide compared to conventional calcium hydroxide. Consistent with the findings of the present study, both the Sireesha and Firas investigations concluded that intracanal medicaments with greater tubule penetration, such as nanoformulations, exhibit superior antibacterial properties.

In a study by Krishnaraj et al. [40], the dentinal penetration depth and antimicrobial efficacy of nano-chlorhexidine were compared with those of a 2% chlorhexidine solution. Similar to previous studies [41] evaluating the penetration capability of intracanal nanoformulations, their findings were consistent with earlier results. In line with the present study, nano-chlorhexidine demonstrated superior antibacterial properties compared to conventional chlorhexidine. According to earlier research [42], although dual and triple antibiotic combinations exhibit strong antibacterial effects, they are often associated with undesirable properties such as cytotoxicity, antibiotic resistance, and tooth discoloration. While the incorporation of silver nanoparticles into intracanal medicaments has been shown to enhance their antibacterial performance and improve the elimination of E. faecalis, tooth discoloration remains a significant concern [37]. Therefore, nanoformulations of chlorhexidine and calcium hydroxide may be preferable over silver nanoparticle-based combinations due to their more favorable esthetic profile. Taken together, the results of previous studies suggest that intracanal nanoformulations are more effective in reducing bacterial biofilm compared to conventional medicaments. Given the significant reduction in CFU observed on day 3, the use of intracanal nano-drugs may be particularly advantageous in clinical scenarios requiring rapid bacterial reduction, such as cellulitis and extensive acute dental abscesses.

## Conclusion

Considering the limitations of this study, MCM-41-based nano-chlorhexidine and Ca(OH)2-PLGA nanoformulations appear to be more effective than conventional medicaments, such as calcium hydroxide and 2% chlorhexidine, for disinfecting dentinal tubules from E. faecalis biofilm, but this effect is not long lasting and further antibacterial agents are needed for long term.

## Conflict of Interest

The author declares that there is no conflict of interest regarding the publication of this article.

## AI Disclosure Statement

During the preparation of this manuscript, the authors used ChatGPT, OpenAI company for language editing, grammar improvement, and liboberry.com for reference management. After its use, the authors thoroughly reviewed, verified, and revised all AI-assisted content to ensure accuracy and originality. The authors take full responsibility for the integrity and final content of the published article.
